# Personality Traits Predict Self-Rated Health (SRH) in Coronary Heart Disease (CHD) Patients and Healthy Controls

**DOI:** 10.3390/healthcare11111645

**Published:** 2023-06-04

**Authors:** Weixi Kang, Antonio Malvaso

**Affiliations:** 1 UK DRI Care Research and Technology Centre, Department of Brain Sciences, Imperial College London, London W12 0BZ, UK; 2 Department of Brain and Behavioral Sciences, University of Pavia, 27100 Pavia, Italy

**Keywords:** personality, Big Five, coronary heart disease, self-rated health

## Abstract

Objective: The objective of the present study is to examine the association between Big Five personality traits and self-rated health (SRH) among individuals with coronary heart disease (CHD), and to compare this relationship with that of healthy control participants, which is of importance as SRH can be a determinant of outcomes. Methods: The current study used data from 566 participants with CHD with a mean age of 63.00 (S.D. = 15.23) years old (61.13% males) and 8608 age- and sex-matched healthy controls with a mean age of 63.87 (S.D.= 9.60) years old (61.93% males) from the UKHLS. The current study used predictive normative modelling approaches, one-sample *t* tests, a hierarchical regression, and two multiple regressions. Results: The current study found that CHD patients have significantly lower Conscientiousness (t(565) = −3.84, *p* < 0.001, 95% C.I. [−0.28, −0.09], Cohen’s d = −0.16) and SRH (t(565) = −13.83, *p* < 0.001, 95% C.I. [−0.68, −0.51], and Cohen’s d = −0.58) scores compared to age and sex-matched healthy controls. Moreover, health status (controls vs. CHD patients) moderated the links between Neuroticism, Extraversion and SRH. Specifically, Neuroticism (b = −0.03, *p* < 0.01, 95% C.I. [−0.04, −0.01]), Openness (b = 0.04, *p* < 0.001, 95% C.I. [0.02, 0.06]), and Conscientiousness (b = 0.08, *p* < 0.001, 95% C.I. [0.06, 0.10]) were significant predictors of SRH in healthy controls, whereas Conscientiousness (b = 0.08, *p* < 0.05, 95% C.I. [0.01, 0.16]) and Extraversion (b = −0.09, *p* < 0.01, 95% C.I. [−0.15, −0.02]) were significant predictors of SRH in CHD patients. Conclusion: Based on the close associations between personality traits and SRH, and the subsequent impact on patient outcomes, the results of this study should be taken into consideration by clinicians and health professionals when developing tailored treatment and intervention programs for their patients.

## 1. Introduction

The most common cause of mortality in the world is coronary heart disease (CHD) [1]. As a result, CHD is the leading cause of morbidity and death in the elderly and places a significant financial strain on the healthcare system [2]. Self-rated health (SRH) is a simple measure of the subjective perception of one’s own health that has good predictive validity, as shown by its association with mortality, morbidity, and functional status [3,4]. Moreover, these associations are still held even after adjustments are made for sociodemographic, clinical, and behavioral risk factors [5].

These findings are being applied to the cardiological field [6,7,8], where it has been found that SRH may be predictive of cardiac events [7,8] and mortality [6]. Particularly, the findings support the use and endorsement of SRH as a supplementary outcome measure in clinical trials. Some have [9] used SRH as an additional part of routine risk assessment in clinical settings [10]. The rationale behind this is that SRH can convey subjective evaluation information that cannot be captured by existing clinical measures [11]. Regarding the association between CHD and SRH, Orimoloye et al. [12] suggested that SRH can be used as an excellent measure of CHD risk. In comparison to the 2013 College of Cardiology/American Heart Association atherosclerotic cardiovascular disease risk score [12], SRH’s combinations with artery calcium score demonstrate similar risk discrimination for CHD and cardiovascular disease events. Indeed, SRH serves as an independent predictor for CHD [13].

However, the incorporation of information into subjective ratings of health, either consciously or subconsciously, has been a topic of debate. Recent research suggests that, in addition to the use of demographic and medical data, individuals’ conscious representation of health complaints, such as fatigue, tiredness, and weakness, which reflect the acknowledged severity of symptoms [14,15,16,17], as well as the awareness of somatic reactions and interoceptive processes which are sensed but not necessarily linked to the aforementioned health complaints and interoceptive processes, such as heartbeat, are two dimensions through which SRH may reflect an individual’s perception [18,19,20]. Additionally, research has shown that the self-perception of health that patients develop about the disease itself is an important determinant of different aspects of recovery, an assertion backed by some theoretical models. For instance, the common-sense model (CSM) put forth by Leventhal et al. [21,22] claims that people consider factors such as perceived controllability through reliance on individual coping mechanisms, experienced symptoms that are attributed to “the illness (identity), beliefs about the factors responsible for it (cause), impact on quality of life and functional capacity (consequences), beliefs about time course and cyclicity (acute/chronic and cyclical timeline), perceived controllability through personal coping behaviours and medical treatment (personal and treatment control), presence of negative emotions related to the illness (emotional representations) and degree of overall understanding of the condition (coherence)” [23]. These theories suggest that the self-perception of health is guided by personal, environmental, and cultural factors, and that these also determine coping strategies. Thus, dispositional factors such as personality, as captured by the Big Five test, can affect self-perception of health. Several cohort studies have reported a negative association between Neuroticism and SRH, while a positive association has been observed between SRH and Extraversion, Openness, Agreeableness, and Conscientiousness [24]. In the context of heart disease, illness representations can predict the chance of in-hospital complications [25], physical functioning and disability after discharge [26], and attendance of rehabilitation programs [27].

Despite their importance, prior research has not investigated the personality factors associated with SRH among individuals with CHD. We wondered if CHD could moderate the associations between personality traits and SRH. Therefore, the current study aims to examine the relationship between Big Five personality traits and SRH in CHD patients, and to compare these findings to the results obtained from healthy controls. This investigation is important because SRH is a crucial determinant of outcomes.

## 2. Methods

### 2.1. Data

Understanding society: the UK Household Longitudinal Study (UKHLS), which has been gathering yearly data from the original sample of UK homes since 1991, was the source of the data utilized in the study [28]. Please refer to https://www.understandingsociety.ac.uk for the details of this study (accessed on 25 August 2022). In Wave 1, all participants initially answered a question about whether they had received a clinical diagnosis of CHD (collected between 2009 and 2010). Next, up to Wave 3, individuals were once more asked if they had just received a CHD diagnosis. Participants who indicated that they had been diagnosed with CHD at Wave 1, Wave 2, or Wave 3 were considered as people with CHD and vice versa. Wave 3 also collected information about personality, demographics, and psychiatric distress items (collected between 2011 and 2012). The patients who had not received a clinical diagnosis of CHD were chosen as controls since they were age and sex matched. Participants with any relevant missing data were also excluded from further analysis. Consequently, there were 8,608 healthy controls with a mean age of 63.87 ± 9.60 years old (61.93% men) and 566 patients with CHD who had a mean age of 63.00 ± 15.23 years (61.13% males). In Table 1, descriptive statistics are shown.

### 2.2. Measures

#### 2.2.1. CHD

Self-reported cardiovascular disease’s veracity has been confirmed, as indicated by its consistency with medical records (e.g., [29]). The question in Wave 1 was “Have you ever been informed that you have any of these disorders by a doctor or other health professional? Heart illness.” Participants in subsequent waves were questioned about whether or not they had recently received a CHD diagnosis.

#### 2.2.2. Personality Traits

The 15-item Big Five Inventory [30], with a Likert scale ranging from 1 (“disagree strongly”) to 5 (“agree strongly”), was used to assess personality. When necessary, scores were reverse-coded. You may obtain the precise set of inquiries that were posed to participants: https://www.understandingsociety.ac.uk/documentation/mainstage/dataset-documentation/term/personality-traits?search_api_views_fulltext= (accessed on 25 August 2022).

#### 2.2.3. SRH

On a scale from 1 (excellent) to 5 (very poor), participants replied to the question, “In general, would you describe your health is...” This particular subjective health measurement has a modest level of reliability (e.g., [31]). When the SRH score was reverse-coded, a higher score indicated greater health.

#### 2.2.4. Control Variables

Age, sex, monthly income, greatest level of education attained, married status, and psychological distress as determined by the GHQ-12 were among the demographic factors [32]. Please refer to Table 1 for the specific coding of these variables.

### 2.3. Analysis

This study employed a predictive normative modeling approach to examine differences in Big Five personality traits and SRH between healthy individuals and those with CHD. The approach involved training six generalized linear models with demographics and psychological distress as the predictors and personality traits and SRH as the predicted variables in healthy controls. The same linear models were then applied to CHD patients to predict their estimated personality trait scores and SRH by using the demographics and psychological distress as predictors. Differences in personality characteristics and SRH between CHD patients and healthy controls were assessed using one-sample *t* tests. This approach gives more advantages than paired-sample *t* tests because it can control for demographic confounders and does not require equal sample sizes. To analyze how personality traits relate to SRH in healthy controls and CHD patients differently, a hierarchical regression was conducted. We performed this by taking age, monthly income, highest educational qualification, marital status, psychological distress, CHD status (healthy controls = 0 and CHD patients = 1), and personality traits, including Neuroticism, Agreeableness, Openness, Conscientiousness, and Extraversion, as well as personality traits by CHD status interactions [33], and feeding them into regression models as predictors, with SRH serving as the predicted variable. In the final step, two multiple regression analyses were performed to examine the relationship between demographic variables, psychological distress, and personality traits as the predictors, and SRH as the outcome variable. The regression analyses were conducted separately for healthy controls and CHD patients. MATLAB 2018a was used as the statistical software.

## 3. Results

**Figure 1:**
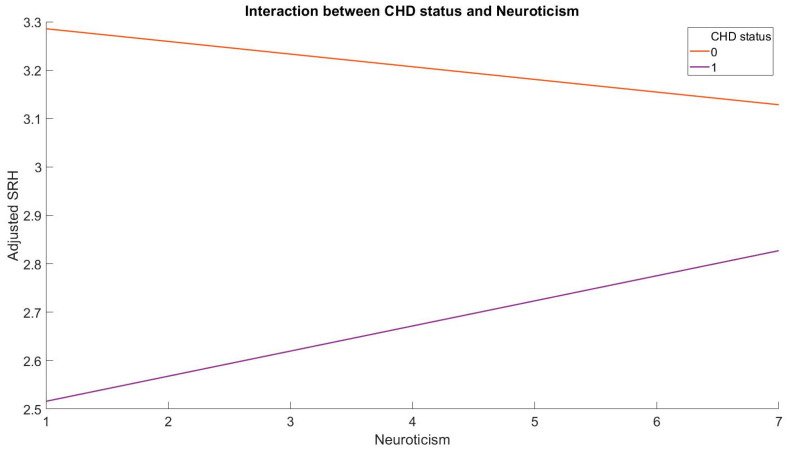
The interaction between Neuroticism and CHD status in predicting SRH.

**Figure 2:**
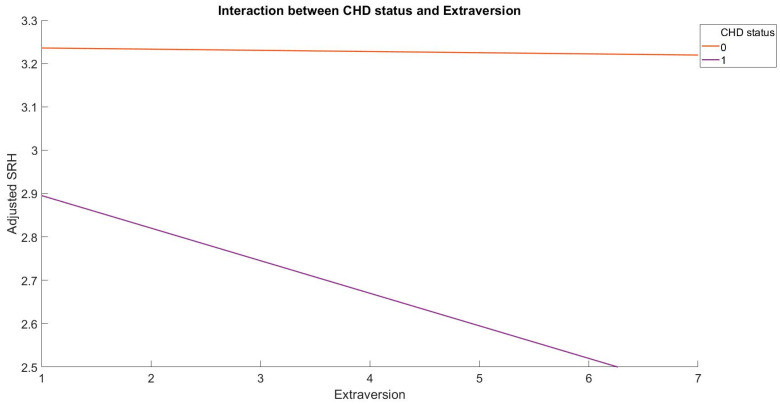
The interaction between Extraversion and CHD status in predicting SRH.

The present investigation aimed to examine the relationship between Big Five personality traits and SRH in patients with coronary heart disease (CHD) and compare it with that of healthy controls. The results indicated that CHD patients had significantly lower Conscientiousness (t(565) = −3.84, *p* < 0.001, 95% C.I. [−0.28, −0.09], Cohen’s d = −0.16) and SRH (t(565) = −13.83, *p* < 0.001, 95% C.I. [−0.68, −0.51], Cohen’s d = −0.58) scores than healthy controls after controlling for demographics. Moreover, significant interactions were observed between Neuroticism (Figure 1; b = 0.08, *p* < 0.01, 95% C.I. [0.02, 0.14]) and Extraversion by CHD status (Figure 2; b = −0.07, *p* < 0.05, 95% C.I. [−0.14, −0.01]), with the overall hierarchical regression model explaining 24.4% of total variances. The multiple regression model accounted for 22.3% (R^2^ = 0.223) of the total variance in SRH among healthy controls, with Neuroticism (b = −0.03, *p* < 0.01, 95% C.I. [−0.04, −0.01]), Openness (b = 0.04, *p* < 0.001, 95% C.I. [0.02, 0.06]), and Conscientiousness (b = 0.08, *p* < 0.001, 95% C.I. [0.06, 0.10]) serving as significant predictors. However, in CHD patients, the regression model accounted for 28.9% (R^2^ = 0.289) of the total variance, with Conscientiousness (b = 0.08, *p* < 0.05, 95% C.I. [0.01, 0.16]) and Extraversion (b = −0.09, *p* < 0.01, 95% C.I. [−0.15, −0.02]) as significant predictors of SRH (Table 2).

## 4. Discussion

The current study’s objective was to examine the relationship between the Big Five personality characteristics and SRH in CHD patients and to compare the findings to those from healthy controls. According to the current study, compared to healthy controls of the same age and sex, CHD patients’ Conscientiousness and SRH scores are considerably lower. Moreover, CHD status moderates the links between Neuroticism, Extraversion and SRH. Specifically, Openness, Neuroticism, and Conscientiousness are significant predictors of SRH in healthy controls, whereas Conscientiousness and Extraversion are significant predictors of SRH in CHD patients.

Big Five personality traits and CHD have not yet been fully studied in the literature, although prior research has revealed that Type A (the tendency of competitive, aggressive, hostile, and pressured of time) [34,35,36,37,38] and Type D (characterized with high negative affectivity combined with high levels of social inhibition) personality are major risk factors for CHD [39,40]. However, less is known about how the Big Five is related to CHD. The current study found that CHD patients are less conscientious, which can be explained by the fact that in general low Conscientiousness scores are linked to lower rates of prosocial and health-promoting activities [41]. Moreover, diseases such as CHD may physically prevent people from being task-focused and doing things thoroughly and orderly rather than being disorganized. However, this seems to contradict another previous study [42], which concluded that individuals with incident CHD have significantly lower Openness and Extraversion scores. This inconsistency may be explained by the small-sized sample in the previous study. The current study also found that CHD patients have poorer SRH with a medium effect size, indicating that CHD patients may consciously know that their health is impaired as a result of CHD. This finding is also consistent with many other previous studies [12,13].

In both healthy controls and CHD patients, we found that Conscientiousness emerges as the personality trait that exhibits a positive correlation with SRH, which is in line with prior research findings. Previous studies have suggested that Conscientiousness is associated with health-promoting practices like physical activity [43], and fewer harmful behaviors, such as smoking and alcohol consumption [44,45,46]. These health-related behaviors may ultimately impact SRH in a positive or negative manner. In addition, Conscientiousness is negatively related to the risk of chronic diseases [47] such as obesity [48]. Few depressive symptoms can be seen over time in people with better SRH [49]. Previous research has also shown that higher Conscientiousness is associated with faster walking speed, better lung function, and stronger grip strength [50], which may result in positive SRH. This connection may also be due to biological causes. In fact, Conscientiousness is linked to improved inflammatory, metabolic, and cardiovascular indicators [50,51], as well as to better cardiorespiratory fitness [52], which may then relate to better SRH.

However, Neuroticism was found to be negatively associated with SRH, and Openness was positively associated with SRH in healthy controls but not in CHD patients, which seems to be consistent with previous studies. Neuroticism has been found to have a negative association with objective health measures, which include behavioral markers like walking speed (e.g., [53,54]) and biological dysfunction [55]. Moreover, Neuroticism is a consistent predictor of poor health outcomes [49]. Furthermore, research suggests that individuals with elevated levels of Neuroticism tend to exhibit a negative bias in their perceptions of the world, leading them to evaluate their own health as being poorer than objective measures would indicate [56]. Moreover, high Openness is associated with more physical activities [43], better physical functions [53,54], and a lower inflammation rate [51], which may then lead to better SRH. CHD patients may be unable to participate in physical activities due to their physical constraints and a higher inflammation rate [57], which may then break the link between Openness and SRH in CHD patients. Extraversion is negatively related to SRH in CHD, but not in healthy controls. This can be explained as, although people with high Extraversion can obtain positive health outcomes through positive emotionality, social relationships, and support [58], which can lead to better SRH, CHD patients generally have lower positive emotionality and fewer social supports [59]. Individuals with high extroversion may be prone to seek out social experiences and rewards in risky behaviors [60] such as substance use [61], which may be especially true when they have chronic conditions such as CHD.

## 5. Limitations

Despite the notable strengths of the present study, such as a robust sample size, utilization of age and sex-matched healthy controls, and rigorous control of sociodemographic variables and psychological distress, a few limitations remain. One limitation of the present study is its cross-sectional design, which precludes the establishment of causal relationships. Future studies should use a longitudinal approach to understand whether personality traits cause better or worse SRH and to determine if SRH causes higher or lower personality trait scores. A second limitation is that the current study relied on self-reported measures [62], which can be subject to bias. The outcomes from the current study should be verified in future research using more objective measures. Predictive normative models may not always generalize well to other populations or settings. Therefore, the model’s predictive performance may be limited to the specific context in which it was developed and may not apply to different populations or settings without appropriate validation. Future studies should focus on other populations as well.

## 6. Conclusions

In sum, the present study aimed to examine the relationship between personality traits and SRH among individuals with CHD, and to compare these associations with those observed in healthy controls. The findings revealed that, in comparison to age- and sex-matched healthy controls, CHD patients reported significantly lower scores on measures of Conscientiousness and SRH. Moreover, CHD status moderated the links between Neuroticism and Extraversion and SRH. Specifically, Openness and Conscientiousness were significant predictors of SRH in healthy controls, whereas Conscientiousness and Extraversion were significant predictors of SRH in CHD patients. There are some implications of the current study as well. As personality traits are directly associated with SRH, which is itself related to outcomes [63], the results of this study should be taken into consideration by clinicians and health professionals when developing tailored treatment and intervention programs for their patients.

## Figures and Tables

**Table 1:** Descriptive statistics for healthy control and CHD patients at Wave 3.

	Healthy Controls (N = 566)		CHD Patients (N = 8608)	
	Mean	S.D.	Mean	S.D.
Age	63.87	9.60	63.00	15.23
Monthly income	1543.92	1525.34	1380.76	1112.09
SRH	3.24	1.13	2.51	1.18
GHQ-12	10.53	5.06	11.86	5.83
Neuroticism	3.25	1.46	3.49	1.50
Agreeableness	5.65	1.05	5.57	1.14
Openness	4.47	1.36	4.33	1.44
Conscientiousness	5.55	1.13	5.32	1.18
Extraversion	4.49	1.37	4.49	1.34
	N	%	N	%
**Sex**				
Male	5331	61.93	346	61.13
Female	3277	38.07	220	38.87
**Highest educational qualification**				
Below college	6397	74.31	441	77.92
College	2211	25.69	125	22.08
**Legal marital status**				
Single	3032	35.32	224	39.58
Married	5576	64.78	342	60.42

**Table 2:** The estimates (b) of multiple regression models for healthy controls and CHD patients by taking demographics, psychological distress, and personality traits as the predictors and SRH as the predicted variable. All numbers are rounded up to two digits.

	Healthy Controls (N = 566)	CHD Patients (N = 8608)
Age	−0.01 ***	−0.02 ***
Sex	0.20 ***	0.29 **
Monthly income	0.00 ***	0.00 ***
Highest educational qualification	0.26 ***	0.14
Legal marital status	0.15 ***	0.07
Psychological distress	−0.08 ***	−0.07 ***
Neuroticism	−0.02 **	0.02
Agreeableness	−0.01	−0.01
Openness	0.04 **	0.02
Conscientiousness	0.08 ***	0.08 *
Extraversion	0.00	−0.09 **
R^2^	0.223	0.289

* *p* < 0.05 ** *p* < 0.01 *** *p* < 0.001.

## Data Availability

Publicly available datasets were analyzed in this study. These data can be found here: https://www.understandingsociety.ac.uk (accessed on 25 August 2022).

## References

[1] Wang H., Naghavi M., Allen C., Barber R.M., Bhutta Z.A., Carter A., Casey D.C., Charlson F.J., Chen A.Z., Coates M.M. (2016). Global, regional, and national life expectancy, all-cause mortality, and cause-specific mortality for 249 causes of death, 1980–2015: A systematic analysis for the Global Burden of Disease Study 2015. Lancet.

[2] Zeller T., Appelbaum S., Kuulasmaa K., Palosaari T., Blankenberg S., Jousilahti P., Salomaa V., Karakas M. (2019). Predictive value of low testosterone concentrations regarding coronary heart disease and mortality in men and women–evidence from the FINRISK 97 study. J. Intern. Med..

[3] DeSalvo K.B., Bloser N., Reynolds K., He J., Muntner P. (2006). Mortality prediction with a single general self-rated health question. J. Gen. Intern. Med..

[4] Idler E.L., Benyamini Y. (1997). Self-rated health and mortality: A review of twenty-seven community studies. J. Health Soc. Behav..

[5] Van der Linde R.M., Mavaddat N., Luben R., Brayne C., Simmons R.K., Khaw K.T., Kinmonth A.L. (2013). Self-rated health and cardiovascular disease incidence: Results from a longitudinal population-based cohort in Norfolk, UK. PLoS ONE.

[6] Bosworth H.B., Siegler I.C., Brummett B.H., Barefoot J.C., Williams R.B., Clapp-Channing N.E., Mark D.B. (1999). The association between self-rated health and mortality in a well-characterized sample of coronary artery disease patients. Med. Care.

[7] Havranek E.P., Lapuerta P., Simon T.A., L’Italien G., Block A.J., Rouleau J.L. (2001). A health perception score predicts cardiac events in patients with heart failure: Results from the IMPRESS trial. J. Card. Fail..

[8] Rutledge T., Linke S.E., Johnson B.D., Bittner V., Krantz D.S., Whittaker K.S., Eastwood J.A., Eteiba W., Cornell C.E., Pepine C.J. (2010). Self-rated versus objective health indicators as predictors of major cardiovascular events: The NHLBI-sponsored Women’s Ischemia Syndrome Evaluation. Psychosom. Med..

[9] Hays R.D., Revicki D. (2011). Assessing Quality of Life in Clinical Trials: Methods and Practice.

[10] Benyamini Y., Gerber Y., Molshatzki N., Goldbourt U., Drory Y. (2014). Recovery of self-rated health as a predictor of recurrent ischemic events after first myocardial infarction: A 13-year follow-up. Health Psychol..

[11] Mavaddat N., Parker R.A., Sanderson S., Mant J., Kinmonth A.L. (2014). Relationship of self-rated health with fatal and non-fatal outcomes in cardiovascular disease: A systematic review and meta-analysis. PLoS ONE.

[12] Orimoloye O.A., Mirbolouk M., Uddin S.I., Dardari Z.A., Miedema M.D., Al-Mallah M.H., Yeboah J., Blankstein R., Nasir K., Blaha M.J. (2019). Association between self-rated health, coronary artery calcium scores, and atherosclerotic cardiovascular disease risk: The multi-ethnic study of atherosclerosis (MESA). JAMA Netw. Open.

[13] Møller L., Kristensen T.S., Hollnagel H. (1996). Self rated health as a predictor of coronary heart disease in Copenhagen, Denmark. J. Epidemiol. Community Health.

[14] Barsky A.J., Cleary P.D., Klerman G.L. (1992). Determinants of perceived health status of medical outpatients. Soc. Sci. Med..

[15] Höfer S., Benzer W., Alber H., Ruttmann E., Kopp M., Schussler G., Doering S. (2005). Determinants of health-related quality of life in coronary artery disease patients: A prospective study generating a structural equation model. Psychosomatics.

[16] Mikolajczyk R.T., Brzoska P., Maier C., Ottova V., Meier S., Dudziak U., Ilieva S., El Ansari W. (2008). Factors associated with self-rated health status in university students: A cross-sectional study in three European countries. BMC Public Health.

[17] Molarius A., Janson S. (2002). Self-rated health, chronic diseases, and symptoms among middle-aged and elderly men and women. J. Clin. Epidemiol..

[18] Craig A.D. (2003). Interoception: The sense of the physiological condition of the body. Curr. Opin. Neurobiol..

[19] Jylhä M. (2009). What is self-rated health and why does it predict mortality? Towards a unified conceptual model. Soc. Sci. Med..

[20] Undén A.L., Andréasson A., Elofsson S., Brismar K., Mathsson L., Rönnelid J., Lekander M. (2007). Inflammatory cytokines, behaviour and age as determinants of self-rated health in women. Clin. Sci..

[21] Leventhal H., Benyamini Y., Brownlee S., Diefenbach M., Leventhal E.A., Patrick-Miller L., Robitaille C. (1997). Illness representations: Theoretical foundations. Percept. Health Illn..

[22] Leventhal H., Nerenz D.R., Steele D.J. (2020). Illness representations and coping with health threats. Handbook of Psychology and Health.

[23] Chiavarino C., Poggio C., Rusconi F., Beretta A.A.R., Aglieri S. (2019). Psychological factors and self-rated health: An observative study on cardiological patients. J. Health Psychol..

[24] Stephan Y., Sutin A.R., Luchetti M., Hognon L., Canada B., Terracciano A. (2020). Personality and self-rated health across eight cohort studies. Soc. Sci. Med..

[25] Cherrington C.C., Moser D.K., Lennie T.A., Kennedy C.W. (2004). Illness representation after acute myocardial infarction: Impact on in-hospital recovery. Am. J. Crit. Care.

[26] Juergens M.C., Seekatz B., Moosdorf R.G., Petrie K.J., Rief W. (2010). Illness beliefs before cardiac surgery predict disability, quality of life, and depression 3 months later. J. Psychosom. Res..

[27] French D.P., Cooper A., Weinman J. (2006). Illness perceptions predict attendance at cardiac rehabilitation following acute myocardial infarction: A systematic review with meta-analysis. J. Psychosom. Res..

[28] University of Essex, Institute for Social and Economic Research (2022). Understanding Society: Waves 1–11, 2009–2020 and Harmonised BHPS: Waves 1–18, 1991–2009.

[29] Barr E.L., Tonkin A.M., Welborn T.A., Shaw J.E. (2009). Validity of self-reported cardiovascular disease events in comparison to medical record adjudication and a statewide hospital morbidity database: The AusDiab study. Intern. Med. J..

[30] John O.P., Donahue E.M., Kentle R.L. (1991). The Big Five Inventory—Versions 4a and 5.

[31] Zajacova A., Dowd J.B. (2011). Reliability of self-rated health in US adults. Am. J. Epidemiol..

[32] Goldberg D., Williams P. (1988). A User’s Guide to the General Health Questionnaire.

[33] Aiken L.S., West S.G. (1991). Multiple Regression: Testing and Interpreting Interactions.

[34] Booth Kewley S., Friedman H.S. (1987). Psychological predictors of heart disease: A quantitative review. Psychol. Bull..

[35] Razzini C., Bianchi F., Leo R., Fortuna E., Siracusano A., Romeo F. (2008). Correlations between personality factors and coronary artery disease: From type A behaviour pattern to type D personality. J. Cardiovasc. Med..

[36] Steptoe A., Molloy G.J. (2007). Personality and heart disease. Heart.

[37] Matthews K.A. (1988). Coronary heart disease and Type A behaviors: Update on and alternative to the Booth-Kewley and Friedman (1987) quantitative review. Psychol. Bull..

[38] Myrtek M. (2001). Meta-analyses of prospective studies on coronary heart disease, type A personality, and hostility. Int. J. Cardiol..

[39] Denollet J., deJonge P., Kuyper A., Schene A.H., van Melle J.P., Ormel J., Honig A. (2009). Depression and Type D personality represent different forms of distress in Myocardial INfarction and Depression—Intervention Trial (MIND-IT). Psychol. Med..

[40] Denollet J., Pederson S., Allan R., Fisher J. (2011). Type D personality in patients with cardiovascular disorders. Heart and Mind: The Practice of Cardiac Psychology.

[41] Lunn T.E., Nowson C.A., Worsley A., Torres S.J. (2014). Does personality affect dietary intake?. Nutrition.

[42] Lee H.B., Offidani E., Ziegelstein R.C., Bienvenu O.J., Samuels J., Eaton W.W., Nestadt G. (2014). Five-factor model personality traits as predictors of incident coronary heart disease in the community: A 10.5-year cohort study based on the Baltimore epidemiologic catchment area follow-up study. Psychosomatics.

[43] Sutin A.R., Stephan Y., Luchetti M., Artese A., Oshio A., Terracciano A. (2016). The five-factor model of personality and physical inactivity: A meta-analysis of 16 samples. J. Res. Personal..

[44] Hakulinen C., Hintsanen M., Munafò M.R., Virtanen M., Kivimäki M., Batty G.D., Jokela M. (2015). Personality and smoking: Individual-participant meta-analysis of nine cohort studies. Addiction.

[45] Kang W. (2022). Personality predicts smoking frequency: An empirical examination separated by sex. Personal. Individ. Differ..

[46] Luchetti M., Sutin A.R., Delitala A., Stephan Y., Fiorillo E., Marongiu M., Masala M., Schlessinger D., Terracciano A. (2018). Personality traits and facets linked with self-reported alcohol consumption and biomarkers of liver health. Addict. Behav..

[47] Weston S.J., Hill P.L., Jackson J.J. (2015). Personality traits predict the onset of disease. Soc. Psychol. Personal. Sci..

[48] Jokela M., Hintsanen M., Hakulinen C., Batty G.D., Nabi H., Singh-Manoux A., Kivimäki M. (2013). Association of personality with the development and persistence of obesity: A meta-analysis based on individual–participant data. Obes. Rev..

[49] Hakulinen C., Elovainio M., Pulkki-Råback L., Virtanen M., Kivimäki M., Jokela M. (2015). Personality and depressive symptoms: Individual participant meta-analysis of 10 cohort studies. Depress. Anxiety.

[50] Sutin A.R., Stephan Y., Terracciano A. (2018). Facets of Conscientiousness and objective markers of health status. Psychol. Health.

[51] Luchetti M., Barkley J.M., Stephan Y., Terracciano A., Sutin A.R. (2014). Five-factor model personality traits and inflammatory markers: New data and a meta-analysis. Psychoneuroendocrinology.

[52] Terracciano A., Schrack J.A., Sutin A.R., Chan W., Simonsick E.M., Ferrucci L. (2013). Personality, metabolic rate and aerobic capacity. PLoS ONE.

[53] Stephan Y., Sutin A.R., Bayard S., Križan Z., Terracciano A. (2018). Personality and sleep quality: Evidence from four prospective studies. Health Psychol..

[54] Stephan Y., Sutin A.R., Bovier-Lapierre G., Terracciano A. (2018). Personality and walking speed across adulthood: Prospective evidence from five samples. Soc. Psychol. Personal. Sci..

[55] Sutin A.R., Stephan Y., Terracciano A. (2019). Personality and metabolic dysfunction in young adulthood: A cross-sectional study. J. Health Psychol..

[56] Sutin A.R., Terracciano A. (2016). Five-factor model personality traits and the objective and subjective experience of body weight. J. Personal..

[57] Patel N.H., Dey A.K., Sorokin A.V., Teklu M., Petrole R., Zhou W., Mehta N.N. (2022). Chronic inflammatory diseases and coronary heart disease: Insights from cardiovascular CT. J. Cardiovasc. Comput. Tomogr..

[58] Roberts B.W., Kuncel N.R., Shiner R., Caspi A., Goldberg L.R. (2007). The Power of Personality: The Comparative Validity of Personality Traits, Socioeconomic Status, and Cognitive Ability for Predicting Important Life Outcomes. Perspect. Psychol. Sci..

[59] Compare A., Zarbo C., Manzoni G.M., Castelnuovo G., Baldassari E., Bonardi A., Callus E., Romagnoni C. (2013). Social support, depression, and heart disease: A ten year literature review. Front. Psychol..

[60] Vollrath M.E., Torgersen S. (2008). Personality types and risky health behaviors in Norwegian students. Scand. J. Psychol..

[61] Kotov R., Gamez W., Schmidt F., Watson D. (2010). Linking “big” personality traits to anxiety, depressive, and substance use disorders: A meta-analysis. Psychol. Bull..

[62] Althubaiti A. (2016). Information bias in health research: Definition, pitfalls, and adjustment methods. J. Multidiscip. Healthc..

[63] Olson K.L., Stiefel M., Ross C., Stadler S., Hornak R., Sandhoff B., Merenich J.A. (2016). Self-rated health among patients with coronary artery disease enrolled in a cardiovascular risk reduction service. Popul. Health Manag..

